# Redo per-oral endoscopic myotomy: direct septotomy and cardiomyotomy with mucosal closure via endoscopic hand suturing

**DOI:** 10.1055/a-2725-7141

**Published:** 2025-11-05

**Authors:** Yuto Shimamura, Sujievvan Chandran, Vicki McGarrigle, Marios Efthymiou, Rhys Vaughan, Osamu Goto

**Affiliations:** 1 Department of Gastroenterology and Hepatology, Austin Health, University of Melbourne, Melbourne, Australia; 2 118084 Department of Gastroenterology, Tokyo Metropolitan Cancer and Infectious Diseases Center Komagome Hospital, Tokyo, Japan

A 58-year-old male with Type II achalasia presented with persistent symptoms three years following a prior peroral endoscopic myotomy (POEM) performed via a posterior approach. Despite multiple sessions of pneumatic balloon dilation, his symptoms persisted, with an Eckardt score of 9. He was therefore referred for a redo POEM.

Preoperative endoscopy revealed a residual septum indicating incomplete myotomy during the initial POEM ( Fig. 1 ), and a barium swallow demonstrated the classic bird-beak appearance.

**Fig. 1:**
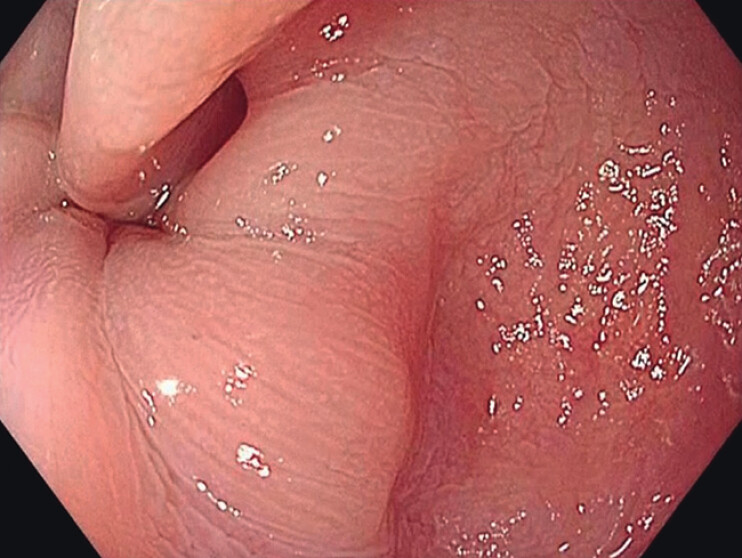
Preoperative endoscopy showing a residual muscular septum, consistent with incomplete myotomy during the initial POEM.

The redo POEM was performed using a GIF-H290T gastroscope (Olympus, Tokyo, Japan) and a Triangle Tip-Jet knife (KD-645L; Olympus) ( Video 1 ). A mucosal incision was made at the 5 o’clock position directly over the septum ( Fig. 2 ), followed by submucosal tunneling extending into the gastric cardia. The distal edge of the previous myotomy was identified, and anatomical landmarks, including penetrating vessels and sling fibers, were used to guide the tunnel into the lesser curvature. Orientation was confirmed using the double-scope method 1 . A complete myotomy was performed starting at the distal edge of the previous myotomy and extended 2 cm into the gastric cardia ( Fig. 3 ). Mucosal entry was closed using absorbable barbed sutures (VLOCL0804; Covidien, Mansfield, MA, USA) via an endoscopic needle holder, SutuArt (FG-260; Olympus) ( Fig. 4 ) 2 . The first stitch was placed distally and advanced proximally, achieving a single-layer, full-thickness closure with six stitches ( Fig. 5 ). A contrast swallow confirmed improved esophageal emptying without any evidence of leakage.

Redo POEM demonstrating direct septotomy and cardiomyotomy with complete mucosal closure via endoscopic hand suturing.Video 1

**Fig. 2:**
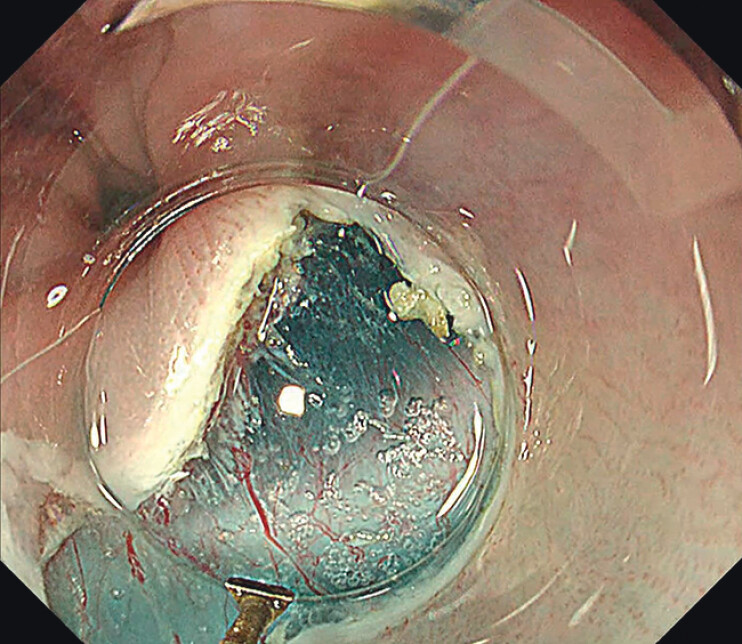
Mucosal incision created at the 5 o’clock position directly over the septum.

**Fig. 3:**
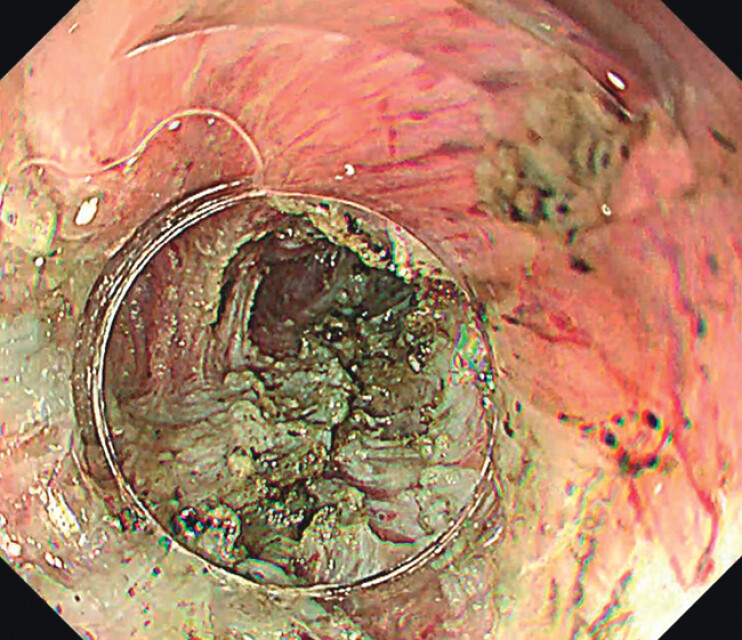
Selective myotomy was performed on the residual muscular septum, extending into the gastric cardia.

**Fig. 4:**
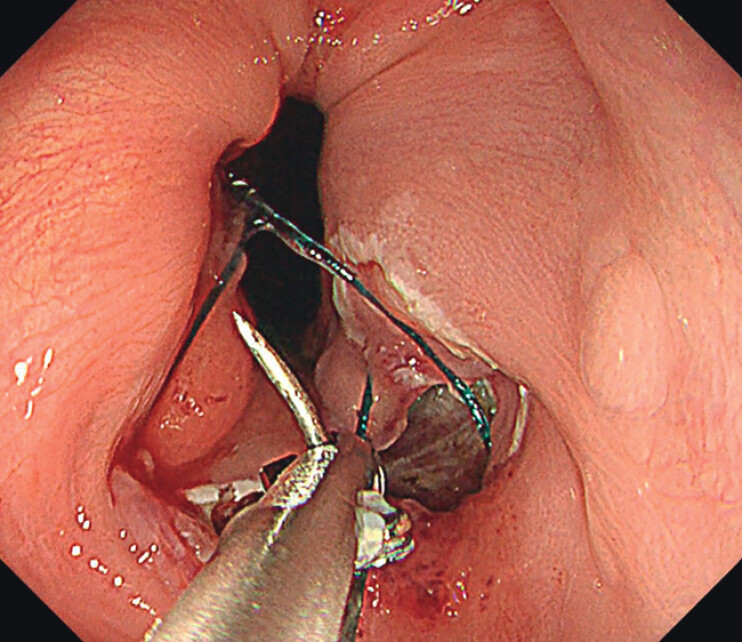
Closure of the mucosal entry using absorbable barbed sutures with an endoscopic needle holder.

**Fig. 5:**
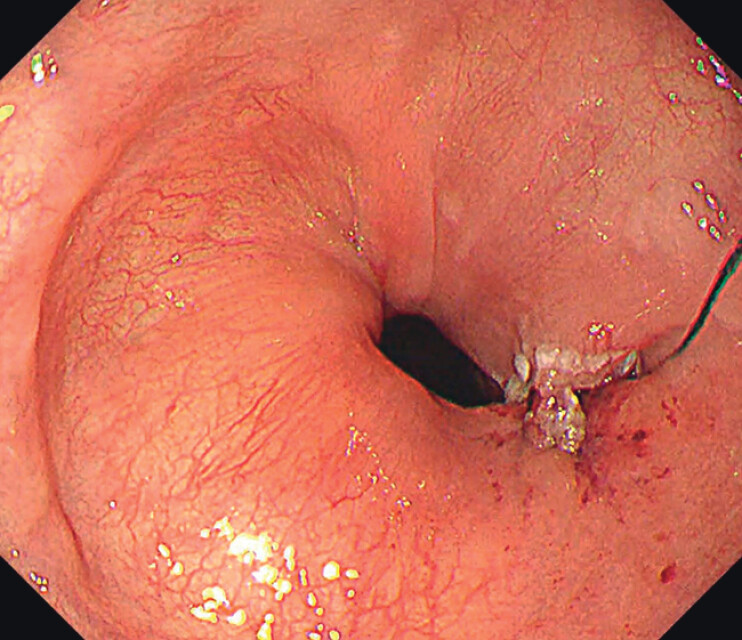
Complete mucosal closure achieved with six stitches.

At two months post-procedure, outcomes were favorable, with an Eckardt score of 1. The patient reported weight gain and marked symptomatic improvement.

While redo POEM has been reported for recurrent achalasia or failed POEM, there is no standardized approach 3 . This case demonstrates that direct septotomy with cardia myotomy is a viable and effective option. Furthermore, complete mucosal closure is critical, highlighting the utility of endoscopic hand suturing.

Endoscopy_UCTN_Code_TTT_1AO_2AP

## Notes

Endoscopy E-Videos https://eref.thieme.de/e-videos
*E-Videos* is an open access online section of the journal *Endoscopy* , reporting on interesting cases and new techniques in gastroenterological endoscopy. All papers include a high-quality video and are published with a Creative Commons CC-BY license. Endoscopy E-Videos qualify for HINARI discounts and waivers and eligibility is automatically checked during the submission process. We grant 100% waivers to articles whose corresponding authors are based in Group A countries and 50% waivers to those who are based in Group B countries as classified by Research4Life (see: https://www.research4life.org/access/eligibility/ ). This section has its own submission website at https://mc.manuscriptcentral.com/e-videos .
